# Supplementary material for: Potential Tamoxifen Repurposing to Combat Infections by Multidrug-Resistant Gram-Negative Bacilli
Source: Pharmaceuticals (Basel). 2021 May 26;14(6):507. doi: 10.3390/ph14060507 (PMC8230278; doi:10.3390/ph14060507)
Supplement: Supplementary file 1 [file pharmaceuticals-14-00507-s001.zip › pharmaceuticals-1229121-supplementary.pdf]

Supplementary Materials

Table S1. Populations of myeloid origin cells, monocytes and neutrophils in the spleen of mice infected with MLD100 of *A. baumannii* ATCC17978, *P. aeruginosa* PAO1 or *E. coli* ATCC25922 strains after 24 h.

	**C11b+ myeloid cells (%)**	**Ly6Chi monocytes (%)**	**Ly6G+ neutrophils (%)**
Control	4.55 ± 0.60	0.39 ± 0.08	0.65 ± 0.16
A. baumannii ATCC 17978	2.71 ± 0.32*	0.07 ± 0.01*	0.40 ± 0.09
P. aeruginosa PAO1	2.66 ± 1.05	0.07 ± 0.02*	0.36 ± 0.17
E. coli ATCC 25922	2.71 ± 0.20*	0.18 ± 0.02*	0.74 ± 0.11

Data are representative of six mice per group, and expressed as mean ± SEM.
*P<0.05: infected mice *vs*. control.

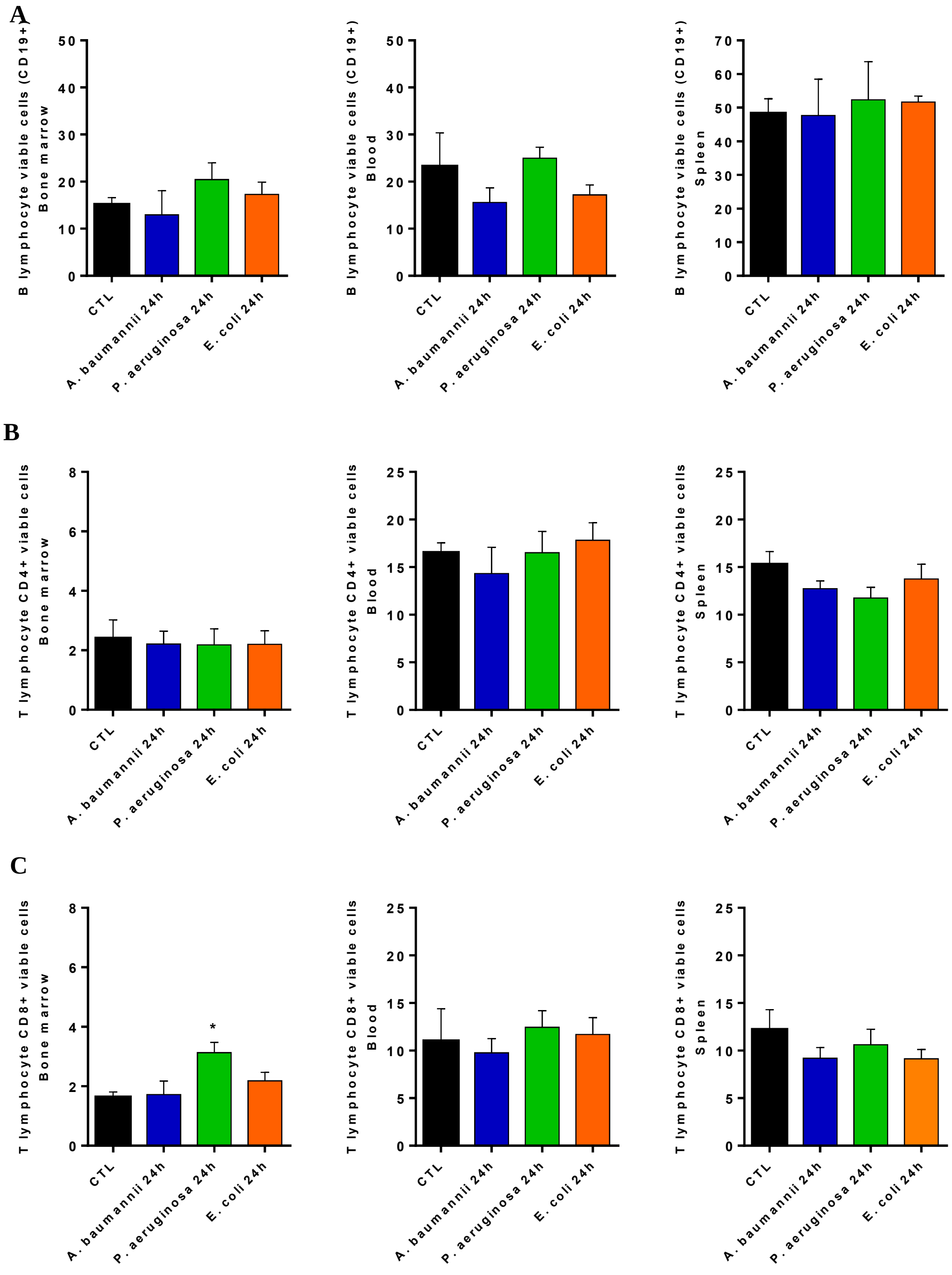

Figure S1. Populations of B lymphocytes and T lymphocytes CD4+ and CD8+ in bone marrow, blood and spleen during bacterial infection. (**A**) B lymphocytes, (**B**) T lymphocytes CD4+ and (**C**) T lymphocytes CD8+ were identified as CD19+, CD4+ and CD8+ by flow cytometry, respectively, in bone marrow, blood and spleen of mice infected with MLD100 of *A. baumannii* ATCC17978, *P. aeruginosa* PAO1 or *E. coli* ATCC25922 strains for 24h. Data are representative of six mice per group, and expressed as mean ± SEM. *P<0.05: infected vs. CTL. CTL: non-infected mice.

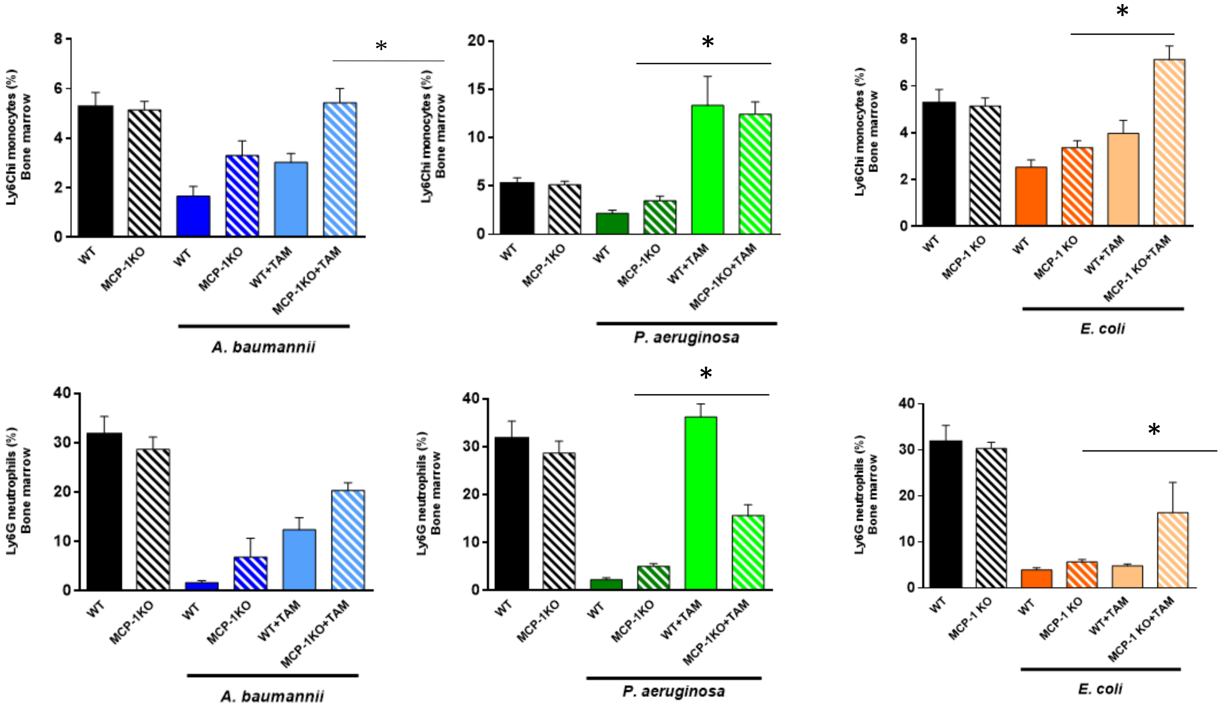

Figure S2. Role of MCP-1 in the bone marrow immune cells migration to blood during bacterial infection. Inflammatory monocytes and neutrophils were identified as CD11b+Ly6Chi and CD11b+Ly6G+ by flow cytometry, respectively, in bone marrow of WT and MCP-1 KO mice infected with MLD100 of *A. baumannii* ATCC17978, *P. aeruginosa* PAO1 or *E. coli* ATCC25922 strains for 24 h. Data are representative of six mice per group, and expressed as mean ± SEM. **P*<0.05: MCP-1 KO+TAM *vs*. MCP-1 KO. WT: wild-type, MCP-1 KO*:* mice lacking MCP-1. CTL: non-infected mice.

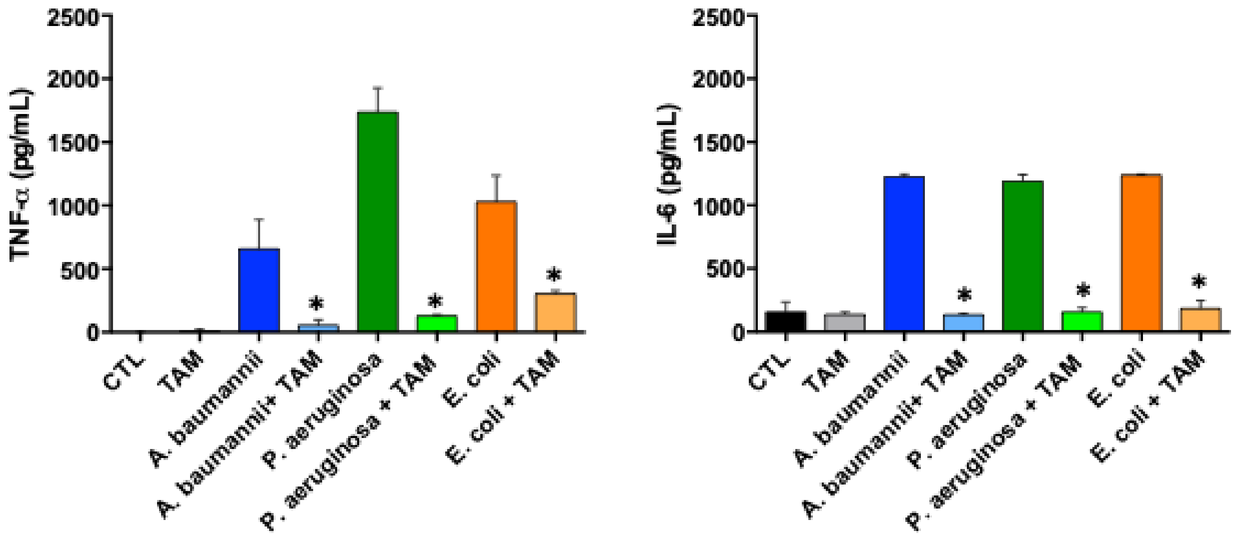

Figure S3. **Tamoxifen effect on cytokines release after bacterial infection.** Mice received tamoxifen (80 mg/kg/d, for 3 days) and infected with minimal lethal dose 100 of *A. baumannii* ATCC17978, *P. aeruginosa* PAO1 or *E. coli* ATCC25922 strains. Twenty-four hours post-infection, (**A**) TNF-α, and (**B**) IL-6 were determined by ELISA in blood of mice. Data are representative of 3 mice per group and are expressed as mean ± SEM. **P*<0.05: infected mice *vs*. infected and treated mice with tamoxifen. CTL: non-infected mice, TAM: tamoxifen.
